# Supplementary material for: Gender difference in ASAS HI among patients with ankylosing spondylitis
Source: PLoS One. 2020 Jul 9;15(7):e0235678. doi: 10.1371/journal.pone.0235678 (PMC7347111; doi:10.1371/journal.pone.0235678)
Supplement: S1 Table — (DOCX) [file pone.0235678.s001.docx]

**Gender difference in ASAS HI among Patients with Ankylosing Spondylitis: a Single Center, Cross-sectional Study**

Hsin-Hua Chen, Yi-Ming Chen, Kuo-Lung Lai, Tsu-Yi Hsieh, Ching-Tsia Lin, Wei-Ting Hung, Yin-Yi Chou, Chih-Wei Tseng, Yi-Da Wu, Chia-Wei Hsieh, Wen-Nan Huang, Yi-Hsing Chen

**Supplemental materials**

Table A. Correlations among ESR, CRP, ASDAS-EAR, ASDAS-CRP, BASDAI, mSASSS, gender and ASAS HI in AS patients

|  | ESR | CRP | ASDAS-ESR | ASDAS-CRP | BASDAI | mSASSS | Gender | ASAS HI |
| --- | --- | --- | --- | --- | --- | --- | --- | --- |
| ESR |  | 0.576^*^ | 0.700^*^ | 0.485^*^ | 0.198^*^ | 0.130^*^ | -0.175^*^ | 0.167^*^ |
| CRP | <0.001^#^ |  | 0.385^*^ | 0.479^*^ | 0.150^*^ | 0.160^*^ | 0.069^*^ | 0.109^*^ |
| ASDAS-ESR | <0.001^#^ | <0.001^#^ |  | 0.843 | 0.781^*^ | 0.078^*^ | -0.228^*^ | 0.487^*^ |
| ASDAS-CRP | <0.001^#^ | <0.001^#^ | <0.001^#^ |  | 0.750^*^ | 0.101^*^ | 0.002^*^ | 0.436^*^ |
| BASDAI | <0.001^#^ | <0.001^#^ | <0.001^#^ | <0.001^#^ |  | 0.018^*^ | -0.103^*^ | 0.580^*^ |
| mSASSS | 0.023^#^ | 0.005^#^ | 0.172^#^ | 0.077^#^ | 0.754^#^ |  |  |  |
| Gender | 0.002^#^ | 0.228^#^ | <0.001^#^ | 0.972^#^ | 0.072^#^ | <0.001^#^ |  |  |
| ASAS-HI | 0.003^#^ | 0.057^#^ | <0.001^#^ | <0.001^#^ | <0.001^#^ | 0.007^#^ | 0.002^#^ |  |

Abbreviations: ESR, erythrocyte sedimentation rate; CRP, C-reactive protein; ASDAS, ankylosing spondylitis disease activity score; BASDAI, Bath ankylosing spondylitis disease activity index.

^*^Pearson correlation coefficient; ^#^P-value.

Table B. Laboratory data and use of medications of patients with AS

|  | Total | Female | Male |  |
| --- | --- | --- | --- | --- |
|  | n = 307 | n = 62 | n = 245 | P-value |
| HLA-B27, n (%) | 300 (97.7) | 60 (96.8) | 240 (98.0) | 0.577 |
| Creatinine, mg/dl | 0.9 ± 0.2 | 0.7 ± 0.1 | 0.9 ± 0.2 | <0.001 |
| Creatinine > 1.4 mg/dl, n (%) | 5 (1.6) | 0 (0.0) | 5 (2.0) | 0.587^*^ |
| ALT, U/L | 26.5 ± 18.5 | 17.7 ± 14.9 | 28.7 ± 18.7 | <0.001 |
| ALT > 40 U/L, n (%) | 43 (14.0) | 2 (3.2) | 41 (16.7) | 0.006 |
| WBC count, /μL | 7283.2 ± 1853.6 | 7152.7 ± 1951.3 | 7316.2 ± 1830.7 | 0.536 |
| Hemoglobin, g/dl | 14.3 ± 1.6 | 12.5 ± 1.3 | 14.7 ± 1.3 | <0.001 |
| RDW, % | 13.4 ± 1.6 | 13.7 ± 1.8 | 13.4 ± 1.5 | 0.079 |
| Platelet count x10^−3^ /μL | 265.4 ± 72.0 | 284.6 ± 82.2 | 260.5 ± 68.4 | 0.018 |
| Current use of biologics | 108 (35.5) | 28 (45.2) | 81 (33.1) | 0.075 |
| Prior use of biologics | 19 (6.2) | 4 (6.4) | 15 (6.1) | 1.000* |
| **Use of medication, n (%)** | | | | |
| Methotrexate | 27 (8.8) | 9 (14.5) | 18 (7.3) | 0.075 |
| Sulfasalazine | 124 (40.4) | 22 (35.5) | 102 (41.6) | 0.378 |
| Hydroxychloroquine | 8 (2.6) | 4 (6.5) | 4 (1.6) | 0.056^*^ |
| Acetaminophen/tramadol | 88 (28.7) | 16 (25.8) | 72 (29.4) | 0.577 |
| NSAID | 269 (87.6) | 54 (87.1) | 215 (87.8) | 0.888 |
| Corticosteroid | 38 (12.4) | 7 (11.3) | 31 (12.7) | 0.771 |

Data were shown as mean ± standard deviation unless specified otherwise.

*Fisher’s exact test.

Abbreviations: AS, ankylosing spondylitis; HLA-B27, human leukocyte antigen-B27; ALT, alanine aminotransferase; RDW, red blood cell distribution width; NSAID, non-steroidal anti-inflammatory drug.

Table C. Comparison of demographic data, clinical characteristics, disease activity related measures and mSASSS between AS patients with ASAS HI≤5 and AS patients with ASAS HI>5

|  | ASAS HI≤5 | ASAS HI>5 |  |
| --- | --- | --- | --- |
|  | n = 194 | n = 113 | P-value |
| Gender |  |  | <0.001 |
| Female | 27 (13.9) | 35 (31.0) |  |
| Male | 167 (86.1) | 78 (69.0) |  |
| Age, year, mean ± SD | 44.6 ± 13.1 | 49.4 ± 13.2 | 0.002 |
| Disease duration, year, mean ± SD | 18.8 ± 11.1 | 23.7 ± 13.2 | 0.001 |
| Smoking | 77 (39.7) | 45 (39.8) | 0.982 |
| **Comorbidities** |  |  |  |
| No. of comorbidities | 1.0 ± 1.1 | 1.4 ± 1.3 | 0.003 |
| Hypertension | 43 (22.2) | 34 (30.1) | 0.122 |
| Diabetes mellitus | 14 (7.2) | 15 (13.3) | 0.080 |
| Hyperlipidemia | 37 (19.1) | 18 (15.9) | 0.489 |
| Hepatitis B | 16 (8.2) | 21 (18.6) | 0.007 |
| Hepatitis C | 3 (1.5) | 5 (4.4) | 0.149^*^ |
| Gout | 10 (5.2) | 8 (7.1) | 0.489 |
| Coronary artery disease | 9 (4.6) | 4 (3.5) | 0.774^*^ |
| Stroke | 1 (0.5) | 0 (0.0) | 1.000^*^ |
| Periodontal disease | 40 (2.6) | 37 (32.7) | 0.018 |
| Osteoporosis | 12 (6.2) | 14 (12.4) | 0.060 |
| **Extra-spinal manifestation** | 95 (49.0) | 70 (61.9) | 0.028 |
| Uveitis | 50 (25.8) | 40 (35.4) | 0.074 |
| Psoriasis | 16 (8.2) | 8 (7.1) | 0.713 |
| Crohn’s disease | 0 (0.0) | 0 (0.0) | - |
| Ulcerative colitis | 0 (0.0) | 1 (0.9) | 0.368^*^ |
| Peripheral arthritis | 45 (23.2) | 42 (37.2) | 0.009 |
| Enthesitis | 28 (14.4) | 29 (25.7) | 0.015 |
| Dactylitis | 5 (2.6) | 4 (3.5) | 0.730^*^ |
| Family history of AS (first or second degree relatives) | 74 (38.1) | 51 (45.1) | 0.229 |
| First degree relatives | 39 (20.1) | 20 (17.7) | 0.606 |
| Second degree relatives | 48 (24.7) | 41 (36.3) | 0.032 |
| ESR, mean ± SD | 10.3 ± 12.2 | 16.2 ± 18.1 | 0.002 |
| CRP, mean ± SD | 0.4 ± 0.7 | 0.8 ± 2.4 | 0.098 |
| ASDAS-ESR, mean ± SD | 1.3 ± 0.6 | 2.0 ± 0.9 | <0.001 |
| ASDAS-CRP, mean ± SD | 1.2 ± 0.7 | 1.9 ± 1.0 | <0.001 |
| BASDAI, mean ± SD | 1.5 ± 1.1 | 3.0 ± 1.6 | <0.001 |
| mSASSS | 15.8 ± 19.9 | 23.5 ± 24.9 | 0.006 |

Data were shown as number (percentage) unless specified otherwise.

*Fisher’s exact test.

Abbreviations: mSASSS, modified Stoke ankylosing spondylitis spinal score; AS, ankylosing spondylitis; ASAS HI, assessment of spondyloarthritis international society health index; SD, standard deviation; ESR, erythrocyte sedimentation rate; CRP, C-reactive protein; ASDAS, ankylosing spondylitis disease activity score; BASDAI, Bath ankylosing spondylitis disease activity index;.

Table D. Univariable and multivariable logistic regression analyses for the associations between gender (female as reference) and items of the ASAS HI in patients with ankylosing spondylitis

|  | Univariable | | Multivariable^*^ | | | | | | | |
| --- | --- | --- | --- | --- | --- | --- | --- | --- | --- | --- |
|  |  |  | Model A | | Model B | | Model C | | Model D | |
| Components of the ASAS HI | OR (95% CI) | P | OR (95% CI) | P | OR (95% CI) | P | OR (95% CI) | P | OR (95% CI) | P |
| Pain sometimes disrupts my normal activities. | 1.07 (0.60–1.91) | 0.82 | 1.43 (0.69–2.97) | 0.339 | 0.85 (0.42–1.74) | 0.654 | 1.07 (0.50–2.27) | 0.861 | 0.92 (0.44–1.92) | 0.819 |
| I find it hard to stand for long. | 0.47 (0.26–0.85) | 0.012 | 0.37 (0.18–0.75) | 0.006 | 0.27 (0.13–0.55) | <0.001 | 0.28 (0.13–0.59) | 0.001 | 0.28 (0.13–0.59) | 0.001 |
| I have problems running. | 0.55 (0.31–0.97) | 0.040 | 0.37 (0.18–0.79) | 0.010 | 0.24 (0.12–0.52) | <0.001 | 0.29 (0.14–0.64) | 0.002 | 0.26 (0.12–0.56) | 0.001 |
| I have problems using toilet facilities. | 0.65 (0.31–1.35) | 0.244 | 0.57 (0.23–1.45) | 0.239 | 0.46 (0.19–1.15) | 0.095 | 0.46 (0.17–1.21) | 0.116 | 0.52 (0.20–1.35) | 0.177 |
| I am often exhausted. | 0.42 (0.24–0.75) | 0.003 | 0.39 (0.20–0.79) | 0.008 | 0.28 (0.14–0.57) | <0.001 | 0.26 (0.12–0.56) | 0.001 | 0.29 (0.14–0.60) | 0.001 |
| I am less motivated to do anything that requires physical effort | 0.48 (0.27–0.84) | 0.010 | 0.35 (0.17–0.72) | 0.004 | 0.25 (0.12–0.50) | <0.001 | 0.26 (0.12–0.54) | <0.001 | 0.26 (0.13–0.55) | <0.001 |
| I have lost interest in sex. | 0.35 (0.17–0.75) | 0.007 | 0.28 (0.10–0.80) | 0.018 | 0.24 (0.08–0.68) | 0.007 | 0.23 (0.08–0.66) | 0.007 | 0.25 (0.09–0.73) | 0.011 |
| I have difficulty operating the pedals in my car. | 1.14 (0.24–5.43) | 0.866 | 0.88 (0.12–6.64) | 0.899 | 0.73 (0.10–5.39) | 0.761 | 0.85 (0.11–6.34) | 0.876 | 0.88 (0.12–6.65) | 0.901 |
| I am finding it hard to make contact with people. | 2.24 (0.65–7.66) | 0.201 | 1.62 (0.41–6.51) | 0.495 | 1.48 (0.37–5.86) | 0.581 | 1.53 (0.38–6.17) | 0.553 | 1.55 (0.39–6.18) | 0.538 |
| I am not able to walk outdoors on flat ground. | 0.55 (0.17–1.86) | 0.338 | 0.62 (0.13–2.94) | 0.550 | 0.57 (0.12–2.67) | 0.473 | 0.54 (0.12–2.49) | 0.425 | 0.52 (0.11–2.36) | 0.393 |
| I find it hard to concentrate. | 0.48 (0.26–0.91) | 0.024 | 0.59 (0.27–1.30) | 0.192 | 0.48 (0.22–1.04) | 0.061 | 0.48 (0.21–1.09) | 0.081 | 0.55 (0.25–1.21) | 0.137 |
| I am restricted in traveling because of my mobility. | 0.57 (0.32–1.02) | 0.056 | 0.61 (0.29–1.28) | 0.191 | 0.46 (0.22–0.94) | 0.034 | 0.54 (0.26–1.14) | 0.107 | 0.51 (0.24–1.07) | 0.073 |
| I often get frustrated. | 0.57 (0.28–1.16) | 0.119 | 0.77 (0.31–1.90) | 0.574 | 0.60 (0.25–1.43) | 0.244 | 0.62 (0.24–1.59) | 0.321 | 0.70 (0.28–1.76) | 0.450 |
| I find it difficult to wash my hair. | 0.24 (0.10–0.57) | 0.001 | 0.10 (0.03–0.43) | 0.002 | 0.08 (0.02–0.33) | <0.001 | 0.09 (0.02–0.38) | 0.001 | 0.08 (0.02–0.33) | 0.001 |
| I have experienced financial changes because of my rheumatic disease. | 1.02 (0.49–2.10) | 0.968 | 0.78 (0.32–1.90) | 0.584 | 0.63 (0.26–1.50) | 0.294 | 0.67 (0.27–1.70) | 0.403 | 0.76 (0.30–1.87) | 0.544 |
| I sleep badly at night. | 0.98 (0.56–1.71) | 0.938 | 1.23 (0.63–2.42) | 0.542 | 0.94 (0.49–1.82) | 0.856 | 1.00 (0.50–2.01) | 0.992 | 1.08 (0.54–2.13) | 0.836 |
| I cannot overcome my difficulties. | 0.39 (0.19–0.82) | 0.014 | 0.65 (0.24–1.78) | 0.400 | 0.42 (0.16–1.11) | 0.080 | 0.55 (0.19–1.58) | 0.266 | 0.52 (0.19–1.15) | 0.211 |

^*^Gender, age, disease duration, smoking, number of comorbidities, extra-spinal manifestations, family history of AS, mSASSS, and disease activity (model A, ASDAS-ESR; model B, ASDAS-CRP; model C, BASDAI+ESR; model C, BASDAI+CRP) were included in the multivariable analyses.

Abbreviations: ASAS HI, assessment of spondyloarthritis international society health index; OR, odds ratio; CI, confidence interval; P, p-value.

Table E. Multiple linear regression analyses for determinants of ASAS HI sum scores in female patients with ankylosing spondylitis

|  | Univariable | | | Multivariable^*^ | | | | | | | |
| --- | --- | --- | --- | --- | --- | --- | --- | --- | --- | --- | --- |
|  |  |  |  | Model A | | Model B | | Model C | | Model D | |
| **Variable** | *B* (95% CI) | P | VIF | *B* (95% CI) | P | *B* (95% CI) | P | *B* (95% CI) | P | *B* (95% CI) | P |
| Age, year | 0.01 (-0.01–0.09) | 0.830 |  | -0.02 (-0.10–0.06) | 0.578 | 0.00 (-0.07–0.08) | 0.905 | -0.01 (-0.09–0.06) | 0.709 | -0.01 (-0.08–0.06) | 0.823 |
| Disease duration, year | 0.07 (-0.01–0.15) | 0.091 |  | 0.06 (-0.04–0.16) | 0.225 | 0.04 (-0.06–0.14) | 0.403 | 0.02 (-0.07–0.12) | 0.612 | 0.02 (-0.07–0.11) | 0.701 |
| Smoking, n (%) | 0.96 (−2.97–4.89) | 0.628 |  | -0.34 (-3.83–3.15) | 0.846 | -0.44 (-4.12–3.24) | 0.810 | -1.21 (-4.59–2.17) | 0.477 | -1.39 (-4.75–1.98) | 0.412 |
| No. of comorbidities | 0.89 (-0.06–1.84) | 0.066 |  | 0.05 (-1.03–1.14) | 0.921 | -0.07 (-1.22–1.08) | 0.907 | 0.22 (-0.79–1.24) | 0.662 | 0.21 (-0.85–1.27) | 0.694 |
| Extra-spinal manifestation | 1.07 (0.27–1.86) | 0.009 |  | -0.27 (-2.01–1.47) | 0.754 | -0.39 (-2.22–1.44) | 0.671 | -0.11 (-1.78–1.56) | 0.894 | -0.05 (-1.73–1.62) | 0.948 |
| Family history of AS | -0.27 (-2.20–1.67) | 0.782 |  | -1.22 (-3.11–0.67) | 0.201 | -0.64 (-2.58–1.30) | 0.511 | -1.17 (-2.94–0.59) | 0.189 | -1.08 (-2.86–0.70) | 0.228 |
| ESR, mm/hour | 0.04 (-0.0003–0.15) | 0.051 |  |  |  |  |  | 0.03 (-0.04–0.09) | 0.405 |  |  |
| CRP, mg/dl | 2.31 (0.52–4.11) | 0.013 | 4 |  |  |  |  |  |  | 0.39 (-1.33–2.11) | 0.654 |
| ASDAS-ESR | 2.76 (1.79–3.73) | <0.001 | 7 | 2.85 (1.78–3.91) | <0.001 |  |  |  |  |  |  |
| ASDAS-CRP | 2.26 (1.40–3.12) | <0.001 | 14 |  |  | 2.20 (1.24–3.16) | <0.001 |  |  |  |  |
| BASDAI | 1.38 (0.95–1.80) | <0.001 | 7 |  |  |  |  | 1.41 (0.93–1.89) | <0.001 | 1.42 (0.92–1.91) | <0.001 |
| mSASSS | 0.04 (-0.05–0.12) | 0.382 |  | 0.02 (-0.06–0.10) | 0.612 | 0.01 (-0.08–0.10) | 0.823 | 0.04 (-0.04–0.12) | 0.282 | 0.04 (-0.04–0.13) | 0.279 |
| AIC |  |  |  | 214 |  | 220 |  | 207.7 |  | 208.3 |  |

^*^Gender, age, disease duration, smoking, number of comorbidities, extra-spinal manifestations, family history of AS, mSASSS, and disease activity (model A, ASDAS-ESR; model B, ASDAS-CRP; model C, BASDAI+ESR; model C, BASDAI+CRP) were included in the multivariable analyses.

Abbreviations: ASAS HI, assessment of spondyloarthritis international society health index; P, p-value; CI, confidence interval; n, number; ESR, erythrocyte sedimentation rate; CRP, C-reactive protein; ASDAS, ankylosing spondylitis disease activity score; BASDAI, Bath ankylosing spondylitis disease activity index; mSASSS, modified Stoke ankylosing spondylitis spinal score, AIC, Akaike information criterion.

Table F. Multiple linear regression analyses for determinants of ASAS HI sum scores in male patients with ankylosing spondylitis

|  | Univariable | | | Multivariable^*^ | | | | | | | |
| --- | --- | --- | --- | --- | --- | --- | --- | --- | --- | --- | --- |
|  |  |  |  | Model A | | Model B | | Model C | | Model D | |
| **Variable** | *B* (95% CI) | P | VIF | *B* (95% CI) | P | *B* (95% CI) | P | *B* (95% CI) | P | *B* (95% CI) | P |
| Age, year | -0.02 (-0.07–0.02) | 0.309 |  | -0.03 (-0.08–0.02) | 0.257 | 0.00 (-0.05–0.05) | 0.953 | -0.02 (-0.06–0.02) | 0.389 | -0.02 (-0.06–0.02) | 0.380 |
| Disease duration, year | 0.07 (0.03–0.10) | <0.001 | 1 | 0.05 (0.01–0.10) | 0.023 | 0.04 (-0.004–0.09) | 0.073 | 0.04 (-0.0003–0.08) | 0.051 | 0.04 (0.00–0.09) | 0.049 |
| Smoking, n (%) | 0.79 (-0.08–1.65) | 0.074 |  | 0.38 (-0.43–1.20) | 0.355 | 0.29 (-0.53–1.11) | 0.480 | 0.48 (-0.27–1.23) | 0.210 | 0.48 (-0.27–1.23) | 0.212 |
| No. of comorbidities | 0.48 (0.13–0.83) | 0.007 | 1 | 0.16 (-0.21–0.53) | 0.397 | 0.17 (-0.20–0.54) | 0.367 | 0.14 (-0.20–0.48) | 0.429 | 0.14 (-0.21–0.48) | 0.436 |
| Extra-spinal manifestation | 1.19 (0.33–2.05) | 0.007 | 1 | 0.28 (-0.52–1.09) | 0.486 | 0.34 (-0.46–1.15) | 0.404 | 0.19 (-0.55–0.92) | 0.618 | 0.17 (-0.56–0.91) | 0.640 |
| Family history of AS | 0.27 (-0.62–1.16) | 0.546 |  | 0.22 (-0.59–1.02) | 0.597 | 0.14 (-0.66–0.95) | 0.728 | 0.12 (-0.62–0.86) | 0.753 | 0.13 (-0.61–0.86) | 0.738 |
| ESR, mm/hour | 0.03 (0.00–0.06) | 0.071 |  |  |  |  |  | -0.01 (-0.03–0.02) | 0.664 |  |  |
| CRP, mg/dl | 0.23 (-0.02–0.48) | 0.068 |  |  |  |  |  |  |  | -0.01 (-0.22–0.20) | 0.900 |
| ASDAS-ESR | 1.88 (1.38–2.38) | <0.001 | 5 | 1.70 (1.19–2.20) | <0.001 |  |  |  |  |  |  |
| ASDAS-CRP | 1.63 (1.17–2.09) | <0.001 | 4 |  |  | 1.53 (1.07–2.00) | <0.001 |  |  |  |  |
| BASDAI | 1.34 (1.09–1.60) | <0.001 | 3 |  |  |  |  | 1.29 (1.03–1.54) | <0.001 | 1.28 (1.02–1.54) | <0.001 |
| mSASSS | 0.04 (0.02–0.05) | <0.001 | 1 | 0.02 (-0.0003–0.04) | 0.055 | 0.02 (-0.002–0.04) | 0.076 | 0.02 (0.01–0.04) | 0.008 | 0.02 (0.01–0.04) | 0.009 |
| AIC |  |  |  | 800 |  | 802 |  | 758 |  | 758 |  |

^*^Gender, age, disease duration, smoking, number of comorbidities, extra-spinal manifestations, family history of AS, mSASSS, and disease activity (model A, ASDAS-ESR; model B, ASDAS-CRP; model C, BASDAI+ESR; model C, BASDAI+CRP) were included in the multivariable analyses.

Abbreviations: ASAS HI, assessment of spondyloarthritis international society health index; P, p-value; CI, confidence interval; n, number; ESR, erythrocyte sedimentation rate; CRP, C-reactive protein; ASDAS, ankylosing spondylitis disease activity score; BASDAI, Bath ankylosing spondylitis disease activity index; mSASSS, modified Stoke ankylosing spondylitis spinal score, AIC, Akaike information criterion.

Table G. Univariable and multivariable logistic regression analyses for determinants of ASAS HI > 5 in female patients with ankylosing spondylitis

|  | Univariable | | | Multivariable^*^ | | | | | | | |
| --- | --- | --- | --- | --- | --- | --- | --- | --- | --- | --- | --- |
|  |  |  |  | Model A | | Model B | | Model C | | Model D | |
| Variable | OR (95% CI) | P | VIF | OR (95% CI) | P | OR (95% CI) | P | OR (95% CI) | P | OR (95% CI) | P |
| Age, year | 1.02 (0.98–1.06) | 0.316 |  | 0.95 (0.88–1.01) | 0.120 | 0.97 (0.91–1.03) | 0.334 | 0.94 (0.87–1.02) | 0.112 | 0.94 (0.87–1.02) | 0.128 |
| Disease duration, year | 1.05 (0.99–1.10) | 0.075 |  | 1.07 (0.98–1.17) | 0.114 | 1.05 (0.97–1.13) | 0.231 | 1.05 (0.96–1.15) | 0.275 | 1.04 (0.95–1.14) | 0.376 |
| Smoking, n (%) | 2.44 (0.24–24.84) | 0.452 |  | 1.17 (0.07–18.45) | 0.912 | 1.68 (0.10–27.02) | 0.716 | 0.56 (0.03–11.98) | 0.709 | 0.51 (0.02–11.58) | 0.675 |
| No. of comorbidities | 1.56 (0.89–2.73) | 0.119 |  | 1.24 (0.51–3.05) | 0.637 | 1.12 (0.48–2.63) | 0.789 | 1.52 (0.59–3.87) | 0.384 | 1.51 (0.59–3.85) | 0.392 |
| Extra-spinal manifestation | 1.20 (0.43–3.32) | 0.725 |  | 0.82 (0.18–3.79) | 0.804 | 0.64 (0.15–2.75) | 0.553 | 0.88 (0.18–4.31) | 0.877 | 0.96 (0.20–4.61) | 0.959 |
| Family history of AS | 1.02 (0.37–2.78) | 0.974 |  | 0.47 (0.10–2.14) | 0.325 | 0.63 (0.15–2.65) | 0.525 | 0.36 (0.07–1.86) | 0.224 | 0.37 (0.07–1.86) | 0.225 |
| ESR, mm/hour | 1.04 (0.99–1.09) | 0.085 |  |  |  |  |  | 1.04 (0.97–1.12) | 0.268 |  |  |
| CRP, mg/dl | 3.51 (0.92–13.48) | 0.067 |  |  |  |  |  |  |  | 1.38 (0.22–8.77) | 0.732 |
| ASDAS-ESR | 5.77 (2.16–15.44) | 0.001 | 7 | 9.99 (2.60–38.37) | 0.001 |  |  |  |  |  |  |
| ASDAS-CRP | 4.29 (1.89–9.72) | 0.001 | 6 |  |  | 4.91 (1.86–12.96) | 0.001 |  |  |  |  |
| BASDAI | 2.71 (1.51–4.86) | 0.001 | 4 |  |  |  |  | 3.85 (1.69–8.74) | 0.001 | 3.88 (1.72–8.75) | 0.001 |
| mSASSS | 1.04 (0.97–1.11) | 0.253 |  | 1.05 (0.94–1.17) | 0.410 | 1.04 (0.95–1.14) | 0.428 | 1.07 (0.95–1.21) | 0.245 | 1.08 (0.97–1.22) | 0.176 |
| AIC |  |  |  | 325 |  | 333 |  | 306 |  | 307 |  |

^*^Gender, age, disease duration, smoking, number of comorbidities, extra-spinal manifestations, family history of AS, mSASSS, and disease activity (model A, ASDAS-ESR; model B, ASDAS-CRP; model C, BASDAI+ESR; model C, BASDAI+CRP) were included in the multivariable analyses.

Abbreviations: ASAS HI, assessment of spondyloarthritis international society health index; OR, odds ratio; CI, confidence interval; P, p-value; VIF, variance inflation fraction; ESR, erythrocyte sedimentation rate; CRP, C-reactive protein; ASDAS, ankylosing spondylitis disease activity score; BASDAI, Bath ankylosing spondylitis disease activity index; mSASSS, modified Stoke ankylosing spondylitis spinal score; AIC, Akaike information criterion.

Table H. Univariable and multivariable logistic regression analyses for determinants of ASAS HI > 5 in male patients with ankylosing spondylitis

|  | Univariable | | | Multivariable^*^ | | | | | | | |
| --- | --- | --- | --- | --- | --- | --- | --- | --- | --- | --- | --- |
|  |  |  |  | Model A | | Model B | | Model C | | Model D | |
| Variable | OR (95% CI) | P | VIF | OR (95% CI) | P | OR (95% CI) | P | OR (95% CI) | P | OR (95% CI) | P |
| Age, year | 1.04 (1.01–1.06) | 0.002 | 3 | 0.99 (0.95–1.03) | 0.609 | 1.01 (0.97–1.05) | 0.568 | 1.00 (0.96–1.04) | 0.840 | 1.00 (0.96–1.04) | 0.902 |
| Disease duration, year | 1.05 (1.02–1.07) | 0.000 | 2 | 1.04 (1.00–1.08) | 0.047 | 1.03 (0.99–1.07) | 0.152 | 1.03 (0.99–1.07) | 0.131 | 1.03 (0.99–1.07) | 0.152 |
| Smoking, n (%) | 1.40 (0.82–2.40) | 0.225 |  | 1.02 (0.53–1.96) | 0.942 | 0.96 (0.50–1.82) | 0.890 | 1.11 (0.56–2.20) | 0.766 | 1.10 (0.56–2.18) | 0.783 |
| No. of comorbidities | 1.45 (1.16–1.80) | 0.001 | 1 | 1.27 (0.96–1.68) | 0.099 | 1.26 (0.95–1.66) | 0.105 | 1.28 (0.95–1.71) | 0.107 | 1.28 (0.95–1.71) | 0.106 |
| Extra-spinal manifestation | 1.84 (1.06–3.19) | 0.030 | 1 | 0.99 (0.52–1.90) | 0.982 | 1.06 (0.56–2.02) | 0.853 | 0.99 (0.50–1.96) | 0.985 | 1.01 (0.51–1.98) | 0.979 |
| Family history of AS | 1.34 (0.78–2.32) | 0.291 |  | 1.47 (0.77–2.80) | 0.247 | 1.38 (0.73–2.63) | 0.324 | 1.59 (0.80–3.15) | 0.188 | 1.58 (0.80–3.14) | 0.192 |
| ESR, mm/hour | 1.02 (1.00–1.04) | 0.029 | 5 |  |  |  |  | 1.01 (0.99–1.03) | 0.471 |  |  |
| CRP, mg/dl | 1.31 (0.97–1.76) | 0.082 |  |  |  |  |  |  |  | 1.06 (0.82–1.38) | 0.652 |
| ASDAS-ESR | 3.45 (2.22–5.35) | <0.001 | 15 | 3.64 (2.23–5.95) | <0.001 |  |  |  |  |  |  |
| ASDAS-CRP | 2.59 (1.81–3.71) | <0.001 | 5 |  |  | 2.92 (1.92–4.45) | <0.001 |  |  |  |  |
| BASDAI | 2.25 (1.75–2.90) | <0.001 | 6 |  |  |  |  | 2.41 (1.81–3.20) | <0.001 | 2.40 (1.80–3.19) | <0.001 |
| mSASSS | 1.02 (1.01–1.04) | <0.001 | 1 | 1.02 (1.00–1.03) | 0.038 | 1.01 (0.99–1.03) | 0.058 | 1.02 (1.01–1.04) | 0.011 | 1.02 (1.01–1.04) | 0.011 |
| AIC |  |  |  | 260 |  | 264 |  | 245 |  | 245 |  |

^*^Gender, age, disease duration, smoking, number of comorbidities, extra-spinal manifestations, family history of AS, mSASSS, and disease activity (model A, ASDAS-ESR; model B, ASDAS-CRP; model C, BASDAI+ESR; model C, BASDAI+CRP) were included in the multivariable analyses.

Abbreviations: ASAS HI, assessment of spondyloarthritis international society health index; OR, odds ratio; CI, confidence interval; P, p-value; VIF, variance inflation fraction; ESR, erythrocyte sedimentation rate; CRP, C-reactive protein; ASDAS, ankylosing spondylitis disease activity score; BASDAI, Bath ankylosing spondylitis disease activity index; mSASSS, modified Stoke ankylosing spondylitis spinal score; AIC, Akaike information criterion.

Table I. Simple linear regression analyses to test the influence of gender on each potential mediators (ESR, CRP, ASDAS-ESR, ASDAS-CRP, BASDAI and mSASSS) in patients with ankylosing spondylitis

| Variable | ESR, mm/hour | | CRP, mg/dl | | ASDAS-ESR | | ASDAS-CRP | | BASDAI | | mSASSS | |
| --- | --- | --- | --- | --- | --- | --- | --- | --- | --- | --- | --- | --- |
|  | *B* (95% CI) | P | *B* (95% CI) | P | *B* (95% CI) | P | *B* (95% CI) | P | *B* (95% CI) | P | *B* (95% CI) | P |
| Gender | -6.49 (-10.59–-2.39) | 0.002 | 0.27 (-0.17–0.71) | 0.228 | -0.46 (-0.68–0.24)) | <0.001 | 0.00 (-0.24–0.25) | 0.972 | -0.38 (-0.80–0.03) | 0.072 | 15.80 (9.85–21.74) | <0.001 |

Abbreviations: ESR, erythrocyte sedimentation rate; CRP, C-reactive protein; ASDAS, ankylosing spondylitis disease activity score; BASDAI, Bath ankylosing spondylitis disease activity index; mSASSS, modified Stoke ankylosing spondylitis spinal score; CI, confidence interval; P, p-value.

Table J. Linear regression analyses to test the influence of gender on ASAS HI sum scores and the influence of gender and the potential mediator with a p-value < 0.05 in Table I on ASAS HI sum scores in patients with ankylosing spondylitis

| Variable | Regressing ASAS HI on gender (equation 2) | | Regressing ASAS HI on gender and each potential mediator (equation 3) | | | | | |
| --- | --- | --- | --- | --- | --- | --- | --- | --- |
|  |  |  | Model A | | Model B | | Model C | |
|  | *B* (95% CI) | P | *B* (95% CI) | P | *B* (95% CI) | P | *B* (95% CI) | P |
| Gender | -1.60 (-2.58–-0.62) | 0.002 | -1.38 (-2.37–-0.39) | 0.006 | -0.65 (-1.54–0.25) | 0.155 | -2.17 (-3.17–-1.17) | <0.001 |
| **Potential mediators** |  |  |  |  |  |  |  |  |
| ESR, mm/hour |  |  | 0.03 (0.01–0.06) | 0.014 |  |  |  |  |
| ASDAS-ESR |  |  |  |  | 2.07 (1.62–2.51) | <0.001 |  |  |
| mSASSS |  |  |  |  |  |  | 0.04 (0.02–0.05) | <0.001 |

Abbreviations: ASAS HI, assessment of spondyloarthritis international society health index; CI, confidence interval; P, p-value; ESR, erythrocyte sedimentation rate; ASDAS, ankylosing spondylitis disease activity score; mSASSS, modified Stoke ankylosing spondylitis spinal score.

Table K. Logistic regression analyses to test the influence of gender on ASAS HI > 5 and the influence of gender and the potential mediator with a p-value < 0.05 in Table I on ASAS HI >5 in patients with ankylosing spondylitis

| Variable | Regressing ASAS HI on gender (equation 2) | | Regressing ASAS HI on gender and each potential mediator (equation 3) | | | | | |
| --- | --- | --- | --- | --- | --- | --- | --- | --- |
|  |  |  | Model A | | Model B | | Model C | |
|  | OR (95% CI) | P | OR (95% CI) | P | OR (95% CI) | P | OR (95% CI) | P |
| Gender | -1.02 (-1.59–-0.45) | <0.001 | -0.89 (-1.48–-0.31) | 0.003 | -0.61 (-1.25–0.02) | 0.058 | -1.45 (-2.07–-0.83) | <0.001 |
| **Potential mediators** |  |  |  |  |  |  |  |  |
| ESR, mm/hour |  |  | 0.02 (0.01–0.04) | 0.008 |  |  |  |  |
| ASDAS-ESR |  |  |  |  | 1.34 (0.94–1.74) | <0.001 |  |  |
| mSASSS |  |  |  |  |  |  | 0.02 (0.01–0.04) | <0.001 |

Abbreviations: ASAS HI, assessment of spondyloarthritis international society health index; CI, confidence interval; P, p-value; ESR, erythrocyte sedimentation rate; ASDAS, ankylosing spondylitis disease activity score; mSASSS, modified Stoke ankylosing spondylitis spinal score.
