# Supplementary material for: Gender difference in ASAS HI among patients with ankylosing spondylitis
Source: PLoS One. 2020 Jul 9;15(7):e0235678. doi: 10.1371/journal.pone.0235678 (PMC7347111; doi:10.1371/journal.pone.0235678)
Supplement: Attachment — Submitted filename: Response-Letter-R2-Gender difference in ASAS HI-20200515.docx [file pone.0235678.s004.docx]

Hsin-Hua Chen

Department of Medical Research

Taichung Veterans General Hospital

Taiwan Boulevard Sect. 4

Taichung, Taiwan 40705, ROC

Tel: 886-4-2359-2525ext4452; Fax: 886-4-2350-3285

E-mail: shc5555@hotmail.com

15 May 2020

Dear Editor

*PLOS ONE*

We wish to thank the reviewer for the helpful comments on our manuscript,

“Gender difference in ASAS HI among patients with ankylosing spondylitis” (Ms. No. PONE-D-19-34404R1)

Below, we have provided point by point responses to these comments, and indicated the revisions in the manuscript.

We hope that you find our revised manuscript acceptable for publication in *PLOS ONE*, and we look forward to hearing from you.

Yours sincerely,

Hsin-Hua Chen

**Review’s Comments to the Author:**

Reviewer #2: The authors have addressed most of my comments. I have several comments:

1. Previous question 5. Where patients excluded if they did not fill out the BASDAI, BASFI or ASDAS questionnaire even though they did fill out the ASAS HI? If yes, please mention this in the methods.

**Author response:** Thanks for your comments. We have mentioned this issue in Line 2-4 of the subsection of Study subjects in the Methods section: ‘Patients were excluded if they did not fill out questionaires required for the assessments of BASDAI, ASDAS or BASFI even though they completed ASAS HI assessments.’

2. Why were patients excluded if the mSASSS was not assessed? The main analysis is about the influence of gender on the ASAS HI and not about the influence of the mSASSS on the ASAS HI. The mSASSS is used as a confounder.

**Author response:** Thanks for your comments. To maximize the validity of the study, we only included patients with available data of major confounders (risk factors for poor health status and presence of data based association between males and females) including mSASSS as mentioned in previous studies (ref 45, 49). Also, as shown in our data, the mSASSS score was quite different between males and females (21.8 ± 23.1 vs. 6.0 ± 11.4, p <0.001). Therefore, we excluded patients without data of mSASSS. We have mentioned this issue in Line 1-11 of the subsection of Potential confounders in the Methods section: ‘Given that ASAS HI was validated to assess health and function in patients with SpA, we only considered the variables that were both shown to be risk factors for function/disability (Chung, Machado, van der Heijde, D'Agostino, & Dougados, 2012; Falkenbach, Franke, & van der Linden, 2003; Forejtova et al., 2008; Landewe, Dougados, Mielants, van der Tempel, & van der Heijde, 2009; Machado et al., 2010; Schiotis et al., 2012; Song, Wang, & Chen, 2017; Ward, Weisman, Davis, & Reveille, 2005; Zhang et al., 2018), health status (Forejtova et al., 2008), or health-related quality of life (Law et al., 2018; van Lunteren et al., 2018) in previous studies and availabe in our data as potential confounder. Therefore, potential confounders in the relationships between gender and ASAS HI include disease activity (Falkenbach et al., 2003; Glintborg et al., 2010; Landewe et al., 2009; Law et al., 2018; Song et al., 2017; van Lunteren et al., 2018), mSASSS (Glintborg et al., 2010; Landewe et al., 2009), age at the index date (Forejtova et al., 2008), disease (symptom) duration (Falkenbach et al., 2003; Forejtova et al., 2008; Law et al., 2018; Ward et al., 2005), smoking history (never, ever/current) (Chung et al., 2012; Ward et al., 2005; Zhang et al., 2018), a family history (first degree or secondary degree relatives) of AS (Forejtova et al., 2008; Ward et al., 2005), number of comorbidities (including hypertension, diabetes mellitus, hyperlipidemia, hepatitis B, hepatitis C, gout, coronary artery disease, stroke, periodontal disease, osteoporosis) (Ward & Kuzis, 2001; Ward et al., 2005), and the presence of any extra-spinal manifestations (i.e., uveitis, psoriasis, inflammatory bowel disease, peripheral arthritis, enthesitis, and dactylitis) (Song et al., 2017; Ward & Kuzis, 2001).’

3. Previous question 11. I am concerned that disease activity might in the causal path between gender and the ASAS HI. This means that disease activity is no longer a confounder. If you correct the analyses for disease activity, the association between gender and ASAS HI might be biased. I understand that the authors would like to investigate the association between ASAS HI with gender and feel that disease activity is an important factor in this association. However, by adding all other factors from the univariable analysis, the effect of disease activity on the association between ASAS HI and gender is blurred by the other factors from the univariable analysis such as smoking. Please provide the results of the analysis between ASAS HI and gender with disease activity and no other factors. This would help to determine if disease activity is in the causal path or not. If not, it also shows the importance and the size of the influence of disease activity.

**Author response:**

(1) Thanks for your great comment! We examined whether or not disease activity measures (i.e., ESR, CRP, ASDAS-ESR, ASDAS-CRP and BASDAI) as well as mSASSS had potential mediation effects for the influence of gender on ASAS HI. We examined the correlations among disease activity measure, mSASSS, gender and ASAS HI (Table A in S1 Table).
(2) Regarding this issue, we added a subsection of Potential mediators in the Methods section: ‘Given that disease activity and structual damage of the spine were the most important risk factors for poor health and function (Landewe et al., 2009; van Lunteren et al., 2018) and were correlated with gender (Table A in S1 Table), we also examined whether or not disease activity measures (i.e., ESR, CRP, ASDAS-ESR, ASDAS-CRP and BASDAI) and mSASSS had potential mediation effects for the influence of gender on ASAS HI.’
(3) We mentioned the statistical method of testing mediation effect in the subsection of Statisical analysis in the Methods section: ‘To examine the potential mediation effects of disease activity measures (i.e., ESR, CRP, ASDAS-ESR, ASDAS-CRP and BASDAI) and mSASSS for the influence of gender on ASAS HI, we examined the three following regression equations: (1) regressing each potential mediators on gender; (2) regressing ASAS HI on gender; (3) regressing ASAS HI on both gender and the potential mediator which had significant association with gender shown in first equation (Baron & Kenny, 1986). The mediation effect was significant if the following four conditions occurred concurrently: (1) gender must affect the potential mediator in the first equation; (2) gender must be shown to influence ASAS HI in the second equation; (3) the potential mediator must affect ASAS HI in the third equation; (4) If all above 3 conditions existed, then the effect of gender on ASAS HI must be less in the third equation than in the second (Baron & Kenny, 1986).
(4) We added 3 tables (Table I-J in S1 Tables) to show the results of statistical analyses for potenial mediation effects. We also added a statement regarding these results in Line 1-9 on page 11 in the last paragraph of the Results section: ‘To test the potential mediation effects of disease activity measures and mSASSS for the influence of gender on ASAS HI, we firstly regress each potential mediator on gender (equation 1). As shown in Table I in S1 Table, gender was significantly associated with ESR, ASDAS-ESR and mSASSS. We then regressed ASAS HI on gender (equation 2) and ASAS HI on both gender and the potential mediator with a p-value < 0.05 in equation 1 (i.e., ESR, ASDAS-ESR and mSASSS) using linear regression analyses and logistic regression analyses. As shown in Table J and Table K, ESR had partial mediation effect given a less effect on ASAS HI in equation 3 than in equation 2, and ASAS-ESR had complete mediation effect given a less effect on ASAS HI in equation 3 than in equation 2 and a non-significant effect of gender on ASAS HI when ASDAS-ESR was controlled in equation 3.’
(5) We added a statement of discussion regarding the issue of mediation effect in Line 12-16 on page 12 in the second paragraph of the Discussion section: ‘We also found that ESR had partial mediation effect and ASDAS-ESR had complete mediation effect for the influence of gender on ASAS HI. Possible explanation was that compared with men, women had a lower hemoglobin level, which may lead to a higher ESR level as well as a worse health status. However, other disease activity measures including CRP, ASDAS-CRP, BASDAI, and mSASSS did not have mediation effects.’

4. Some important features are now missing from Table 1 as they were removed. Please do not remove HLA-B27 status from Table 1 as it is an important SpA feature and the number of patients on NSAIDs and the number of patients on biologicals to get an idea about the study population.

**Author response:** Thanks for your comments. We have added the data of HLA-B27, the number of patients on NSAIDs and the number of patients on biologics in Table 1.

5. Are the first seven variable of Table 2 measured as part of the ASDAS or BASDAI?

**Author response:** Yes. We have marked ASDAS related measures with ‘^*^’ and BASDAI related measures with ‘^#^’ immediately after the variables of Table 2, and mentioned this information in the footnote of Table 2: ‘^*^ASDAS related measures. ^#^BASDAI related measures.’
